# Beyond Rhetoric: A Response to Recent Commentaries

**DOI:** 10.34172/ijhpm.10022

**Published:** 2026-06-08

**Authors:** Eivind Engebretsen, Mona Baker

**Affiliations:** Centre for Sustainable Healthcare Education, Faculty of Medicine, University of Oslo, Oslo, Norway.

 We are grateful for the six commentaries on our editorial. The responses by Anonymous,^1^ BlackDeer,^2^ Ge,^3^ Muller,^4^ Orr and Zielinska,^5^ and Veronese et al^6^ extend and sharpen the arguments we advanced, pressing them in directions we had not fully pursued. What follows takes up three lines of inquiry that emerge with particular force from that exchange: the inadequacy of decolonizing rhetoric in the face of ongoing genocide; the politics of knowledge production and the coloniality of global health; and the demands of moral and political clarity.

 Those questions have only become more urgent. Our editorial was written in early 2024, and the scale of the catastrophe in Gaza has since intensified to a degree that renders even the most urgent language inadequate. The ongoing genocide has laid bare the active complicity of global health institutions in sustaining colonial structures. Several commentators press this point home. Anonymous^1^ situates our editorial within a longer history of the exceptionalization of Palestine in global health, in which the political root causes of ill health—settler colonialism, apartheid, racism—are systematically rendered “too political” and Palestinian health is reduced to the humanitarian realm. This has created a field that, as Anonymous writes, excels at enriching academic portfolios through “conflict zone expertise” while scrupulously avoiding any identification of perpetrators and enablers. Veronese et al^6^ emphasize this point further, arguing that global health is actively complicit in settler colonial structures treating Gaza as “a passive site for intervention, rather than a place where health systems are systematically dismantled by colonial power.”

## The Coloniality of Global Health Knowledge

 The politics of knowledge production receives sustained attention across the commentaries. BlackDeer^2^ grounds our argument in the broader framework of coloniality—specifically the coloniality of knowledge—drawing on Quijano and Tuhiwai Smith to show how hierarchical knowledge production in global health insulates those at the top and marginalizes precisely the voices and experiences that ought to be foundational. The positivist distance that characterizes much global health research – its preference for measurement, epidemiological abstraction, and procedural neutrality is a colonial habit that flattens the depth and specificity of lived suffering under occupation.

 Muller^4^ insists on sensitivity to the specific historical and socio-political conditions through which settler colonialism operates in different localities. Drawing on interdisciplinary scholarship on Indigenous health in Canada, she cautions against broad characterizations of settler colonialism as “an almost faceless, insidious force,” arguing that context-specific analysis centering Indigenous voices and local knowledge is essential if the concept is to generate actionable strategies. Our editorial was inevitably written at a level of abstraction shaped by its scope and form; Muller’s call to de-centre Eurocentric frameworks and presumed universality, and to create space for grassroots understandings, bears directly on the action-to-knowledge strategy we proposed.

 Ge^3^ draws out the territorial scope of this argument, tracing it from Palestine to occupied Turtle Island (Canada) and demonstrating that the imbrication of settler colonialism and health is not geographically contained. The concept of liberatory or revolutionary medicine—developed by practitioners including Abu Sittah and Gilbert—offers a more honest and politically grounded framework than the language of “global health,” whose very universalism conceals its colonial origins. The first step is to contextualize and historicize rather than depoliticize; meaningful solidarity—not benevolent intervention—must define our collective response.

## On Moral Clarity and the Question of October 7

 Orr and Zielinska^5^ raise a challenge that requires a direct response. They argue that the discourse of decolonization, including in our editorial and the commentaries that followed it, systematically overlooks the suffering caused by the October 7 attacks. They contend that the refusal to acknowledge this trauma will deepen Israeli resentment and obstruct the path to a sustainable solution.

 We do not dismiss this concern. The killing and traumatization of Israeli and international civilians on October 7 caused real and irreversible harm that should be acknowledged as such. But October 7 did not happen in a vacuum. “It is not as if there was peace practiced by Israel before that date,” as Spivak put it in a video recorded in solidarity with Palestinians in February 2024.^7^ We also note, however, that the specific purpose of our editorial—and of this exchange—is to examine the failures of global health institutions and decolonizing discourse in responding to an ongoing genocide. The editorial was never intended as a comprehensive account of all parties’ suffering in the region. The argument that attending to Palestinian suffering requires equal attention to all dimensions of Israeli and Palestinian violence simultaneously is itself a discursive move that has consistently been used to dilute and delay a reckoning with colonial accountability.

 We do, however, welcome Orr and Zielinska’s call to distinguish between the general critique of depoliticized healthcare and the specific application of the settler colonialism framework. Their observation that international health organizations have increasingly, if insufficiently, named Israel as aggressor—a development that postdates our editorial—is welcome. Orr and Zielinska also point to the chronic underfunding of World Health Organization (WHO) and other international organizations by powerful states as a structural constraint on their capacity for action, and note the withdrawal of the United States from WHO in January 2025 as a further and worrying manifestation of this problem.

## Towards a Decolonial Praxis

 What these commentaries make undeniable is what we sought to demonstrate in the original editorial: the rhetorical adoption of decolonization in global health has functioned as a “settler move to innocence”^8^ – allowing institutions and scholars to perform commitment to justice while remaining structurally embedded in the very systems that perpetuate colonial violence. BlackDeer^2^ demonstrates, following Freire, that decolonial praxis is neither purely intellectual nor purely activist but requires the dynamic interplay of both. Global health now faces the question of whether it possesses the political will – and sufficient independence from the funders, governments, and epistemic gatekeepers that shape it – to move from acknowledgement to action.

 The words of Lilla Watson, cited by Ge,^3^ name the challenge precisely: “If you have come here to help me, you are wasting your time. But if you have come because your liberation is bound up with mine, then let us work together.” Global health cannot meet that challenge as an external observer who may or may not choose to intervene. Its authority, funding, and epistemological frameworks are themselves produced within the structures of colonial violence. Recognizing that imbrication is the precondition for contributing meaningfully to collective liberation.

## Disclosure of artificial intelligence (AI) use

 Standard Microsoft writing assistance tools were used to improve grammar and language clarity in some sections of the manuscript.

## Ethical issues

 Not applicable.

## Conflicts of interest

 Authors declare that they have no conflicts of interest.

## References

[1] Anonymous Anonymous (2024). Are burned babies and mass graves a global health crisis? What does decolonization got to do with it? Comment on “The rhetoric of decolonizing global health fails to address the reality of settler colonialism: Gaza as a case in point?”. Int J Health Policy Manag.

[2] BlackDeer AA (2024). Countering coloniality in global health: Comment on “The rhetoric of decolonizing global health fails to address the reality of settler colonialism: Gaza as a case in point. ” Int J Health Policy Manag.

[3] Ge Y (2025). Palestine is freeing us all before Palestine is free: Comment on “The rhetoric of decolonizing global health fails to address the reality of settler colonialism: Gaza as a case in point. ” Int J Health Policy Manag.

[4] Muller MK (2024). Centering local knowledge to address the imbrication of settler colonialism and global health: Comment on “The rhetoric of decolonizing global health fails to address the reality of settler colonialism: Gaza as a case in point. ” Int J Health Policy Manag.

[5] Orr Z, Zielinska AC (2025). Depoliticization, colonialism, and the imperative to disrupt denial: Comment on “The rhetoric of decolonizing global health fails to address the reality of settler colonialism: Gaza as a case in point. ” Int J Health Policy Manag.

[6] Veronese G, Kagee A, Abu Jamei Y (2024). Confronting the colonial roots of global health inequities in Gaza: Comment on “The rhetoric of decolonizing global health fails to address the reality of settler colonialism: Gaza as a case in point. ” Int J Health Policy Manag.

[7] Spivak GC. The Colonizer’s Violence Only Generates Violence. Recorded Message of Solidarity With the Palestinian People [Video]. YouTube; 2024. https://www.youtube.com/watch?v=liB2mr9XMp0. Accessed May 18, 2026.

[8] Tuck E, Yang KW (2012). Decolonization is not a metaphor. Decolonization: Indigeneity, Education & Society.

